# Initiation of Supporting Cell Activation for Hair Cell Regeneration in the Avian Auditory Epithelium: An Explant Culture Model

**DOI:** 10.3389/fncel.2020.583994

**Published:** 2020-11-12

**Authors:** Mami Matsunaga, Tomoko Kita, Ryosuke Yamamoto, Norio Yamamoto, Takayuki Okano, Koichi Omori, Satoko Sakamoto, Takayuki Nakagawa

**Affiliations:** ^1^Department of Otolaryngology, Head and Neck Surgery, Graduate School of Medicine, Kyoto University, Kyoto, Japan; ^2^Medical Innovation Center, Kyoto University, Kyoto, Japan

**Keywords:** atoh1, basilar papilla, regeneration, hair cell, supporting cell, transdifferentiation, type 1 interferon

## Abstract

Sensorineural hearing loss is a common disability often caused by the loss of sensory hair cells in the cochlea. Hair cell (HCs) regeneration has long been the main target for the development of novel therapeutics for sensorineural hearing loss. In the mammalian cochlea, hair cell regeneration is limited, but the auditory epithelia of non-mammalian organisms retain the capacity for hair cell regeneration. In the avian basilar papilla (BP), supporting cells (SCs), which give rise to regenerated hair cells, are usually quiescent. Hair cell loss induces both direct transdifferentiation and mitotic division of supporting cells. Here, we established an explant culture model for hair cell regeneration in chick basilar papillae and validated it for investigating the initial phase of hair cell regeneration. The histological assessment demonstrated hair cell regeneration *via* direct transdifferentiation of supporting cells. Labeling with 5-ethynyl-2′-deoxyuridine (EdU) revealed the occurrence of mitotic division in the supporting cells at specific locations in the basilar papillae, while no EdU labeling was observed in newly generated hair cells. RNA sequencing indicated alterations in known signaling pathways associated with hair cell regeneration, consistent with previous findings. Also, unbiased analyses of RNA sequencing data revealed novel genes and signaling pathways that may be related to the induction of supporting cell activation in the chick basilar papillae. These results indicate the advantages of our explant culture model of the chick basilar papillae for exploring the molecular mechanisms of hair cell regeneration.

## Introduction

The mammalian cochlea cannot regenerate hair cells (HCs) after birth (Bohne et al., 1976; Hawkins et al., 1976; Oesterle et al., 2008). Recent studies have revealed that supporting cells (SCs) are capable of generating new HCs *in vitro* (White et al., 2006; Oshima et al., 2007; Sinkkonen et al., 2011). Furthermore, neonatal mammalian cochlear SCs were found to be capable of HC regeneration through direct transdifferentiation and mitotic division under certain conditions (Cox et al., 2014), similar to the avian basilar papilla (BP) SCs. Direct transdifferentiation of SCs can be induced by genetic or pharmacological inhibition of Notch signaling (Yamamoto et al., 2006; Doetzlhofer et al., 2009) or by ectopic expression of Atoh1 (Zheng and Gao, 2003; Kelly et al., 2012; Liu et al., 2012). However, studies on adult animals have shown only limited recovery of hearing ability (Mizutari et al., 2013; Tona et al., 2014). More recently, manipulation of MYC and NOTCH induced HC regeneration *via* SC proliferation in the adult mice (Shu et al., 2019), and *Hes1* modulation in adult guinea pigs resulted in HC restoration (Du et al., 2018). In contrast to mammals, the regenerative capacity of avian BPs is robust and capable of restoring cellular patterning and function (Saunders and Salvi, 2008; Saunders, 2010). Moreover, the potential for HC regeneration is present in the BP throughout the life of the animal, but in an undamaged animal, no spontaneous replacement of HCs occurs in the BP. Previously, analyses of transcriptomic profiles during BP development focused on major signaling pathways, including Notch (Daudet and Lewis, 2005; Daudet et al., 2007; Thiede et al., 2014; Petrovic et al., 2014), fibroblast growth factor (FGF; Bermingham-McDonogh et al., 2001; Jacques et al., 2012a), and Wnt signaling (Sienknecht and Fekete, 2008; Munnamalai et al., 2017). However, there is limited information regarding the molecular pathways and their interactions during HC regeneration in chick BPs compared to that in the zebrafish (Kniss et al., 2016; Denans et al., 2019). More recently, a road map of molecular events during the development of mouse cochlear sensory epithelia has been reported using single-cell ribonucleic acid (RNA) sequencing (RNA-seq; Kolla et al., 2020). These findings provide valuable information for the development of novel strategies for the promotion of HC regeneration in adult mammalian cochleae.

In the avian BP, no HC replacement by SCs has been observed under homeostatic conditions. Once HC loss is induced, the regenerative process is initiated immediately. There are two modes for HC regeneration in the avian BP: direct transdifferentiation of SCs and division of SCs, followed by differentiation into HCs. The former is the predominant process for HC regeneration (Stone and Cotanche, 2007) and can also be induced in adult mammalian cochleae, although with limited capacity (Hori et al., 2007; Mizutari et al., 2013; Tona et al., 2014). On the other hand, in the lateral line of the zebrafish, the primary route for HC regeneration is the mitotic regeneration of SCs (Kniss et al., 2016; Denans et al., 2019). Hence, understanding the precise mechanisms for HC regeneration in chick BPs, especially direct transdifferentiation of SCs, may contribute to a better understanding of the molecular and cellular pathways involved in the regenerative potential of mammalian HCs.

Our ultimate goal is to explore novel strategies for inducing SC activation for HC regeneration in mammalian cochleae. Therefore, we focused on the signals that trigger SC activation in the initial phase of HC regeneration in chick BPs by using an explant culture model for HC regeneration after ototoxic insult. Explant culture systems of chick BPs have been employed for decades to figure out mechanisms for HC regeneration (Oesterle et al., 1993; Stone et al., 1996; Warchol and Corwin, 1996). We examined the temporal and spatial characteristics of the cellular events using our explant culture model. Based on the time course of cellular events in cultured BPs, we defined specific time points for sampling and performed bulk RNA-seq to characterize the initial phase of HC regeneration.

## Materials and Methods

### Animals

Post-hatched 1-day-old male Momiji (*Gallus domesticus*) chicks were purchased from Goto Furan-Jo (Gifu, Japan) and placed in the experimental room, which was maintained at a temperature of approximately 23°C, in the box in which they were transported, less than 3 h after delivery. All animal procedures were performed following the National Institutes of Health (NIH; Bethesda, MD, USA) Guide for the Care and Use of Laboratory Animals (NIH Publications No. 8023, revised 1978) and were approved by the Animal Research Committee of Kyoto University Graduate School of Medicine (Med Kyo 20133).

### BP Explant Culture

The BP explant culture was performed based on previous studies (Stone et al., 1996; Shang et al., 2010; Wan et al., 2020). The middle ear cavity was opened, followed by the extirpation of the cochlear duct. Cochlear ducts were placed in ice-cold sterile medium 199 (catalog #11150059, Thermo Fisher Scientific, Waltham, MA, USA). The tegmentum vasculosum was carefully removed from the cochlear duct. The remaining tissue containing the BP was cultured in a free-floating manner (submerged in the medium) in 500 μl Dulbecco’s modified Eagle’s medium with 4.5 g/l glucose (catalog #08459-35, Nacalai Tesque, Kyoto, Japan) supplemented with 1% fetal bovine serum (catalog #12389802, Thermo Fisher Scientific, Waltham, MA, USA) in 48-well plates (Iwaki Co. Limited, Tokyo, Japan). Culture media were changed every other day. The following antibiotics (Sigma–Aldrich, St. Louis, MO, USA) were also added to culture media: penicillin only, or penicillin/streptomycin (SM; 200 penicillin units per ml and 78 μM streptomycin, catalog #P4333). At the end of SM exposure, SM was washed out by changing the medium twice. For cell proliferation assay, BP explants were treated with 10 μM EdU during the whole culture duration (catalog #C10340, Thermo Fisher Scientific, Waltham, MA, USA). For pharmacological inhibition of the Janus kinase (JAK)/signal transducer and activator of transduction (STAT) signaling pathway, 10 μM ruxolitinib phosphate (catalog #CS-0326, Chem Scene LLC, Monmouth Junction, NJ, USA) was added to the media. The concentration of ruxolitinib phosphate was determined according to a previous publication (Febvre-James et al., 2018).

### Immunohistochemistry

Immunofluorescence analyses were performed on whole-mount preparations or frozen sections of the chick cochlea duct. After fixation with 4% paraformaldehyde in phosphate-buffered saline (PBS) for 15 min at room temperature, dehydration was performed using 15% sucrose with 0.2 mM ethylenediaminetetraacetic acid in PBS overnight followed by 30% sucrose with 0.2 mM ethylenediaminetetraacetic acid at 4°C for the frozen section, as previously described (Bermingham-McDonogh et al., 2001). Cryostat-cut sections (10 μm in thickness) of Optimal Cutting Temperature (catalog #4583, OCT, Sakura Finetek Japan, Co., Ltd., Tokyo, Japan)-embedded frozen samples were mounted directly onto MAS-coated slides (catalog #SMAS-01, Matsunami Glass Inc., Osaka, Japan). The ensemble of sections collected from three to four BPs was distributed to almost 20 slides (7–9 sections per slide) for each experiment. Each slide contained a reliable representation of the entire length of the BP in a serial manner, which provided uniformity of treatment for cross-sections throughout the cochlear length.

Both the whole mount and section samples were incubated in blocking solution (1% bovine serum albumin and 5% normal goat serum in PBS) for 30 min at room temperature. Samples were incubated with primary antibodies in a blocking solution containing 0.2% Triton X-100 (Nacalai Tesque) overnight at 4°C, followed by incubation with the corresponding secondary antibodies for 1 h at room temperature. Mouse anti-myosin VIIa (2 μg/ml; catalog #138-1, Developmental Studies Hybridoma Bank, IA, USA) and rabbit anti-Sox2 (1:500; catalog #AB5603, Sigma–Aldrich, St. Louis, MO, USA) were the primary antibodies used, and Alexa Fluor 488-, 546-, or 647-conjugated anti-mouse or anti-rabbit IgG antibodies (Thermo Fisher Scientific, Waltham, MA, USA) were the secondary antibodies used. 4′,6-diamidino-2-phenylindole (DAPI, catalog #D1306, Thermo Fisher Scientific, Waltham, MA, USA) was used for nuclear counterstaining. After several washes with PBS, samples were mounted using Fluoromount-G (catalog #00-4958-02, Southern Biotech, Birmingham, AL, USA).

EdU staining was performed according to the Click-iT imaging kit instructions (catalog #C10340, Thermo Fisher Scientific, Waltham, MA, USA) after staining for myosin VIIa and Sox2. As regards the whole-mount preparation for EdU staining, the tectorial membrane was removed by treatment with 100 μg/ml subtilisin (catalog #P8038, Sigma–Aldrich, St. Louis, MO, USA) for 10 min at room temperature to expose the sensory epithelia before fixation. Counterstaining with DAPI was performed as the last step of EdU staining.

All fluorescence images were obtained using a TCS-SPE confocal microscope (equipped with 40×/1.15 objective, Leica Microsystems, Wetzlar, Germany). For the whole mount samples, the area around 40% of the distal end of each of the BP samples was used for quantitative analyses. Optical sections in the *xy*-field (“*z*-sections”) were imaged and recorded at 4-μm intervals, with the span adjusted to include the HC and SC layers in the *xy*-field of view. The total BP length between the proximal portion and the distal portion was calculated using ImageJ software (NIH)^1^.

### *In situ* Hybridization

Frozen section samples for *in situ* hybridization (ISH) were prepared similarly to those for immunohistochemistry, with the following amendments: duration of fixation (4°C for 3–4 h), higher ethylenediaminetetraacetic acid concentration (333 mM) in 30% sucrose, and thicker sections (14 μm in thickness). All ISH experiments were performed using the RNAscope 2.5 HD Duplex Detection Kit (catalog #322430, Advanced Cell Diagnostics Inc., Hayward, CA, USA) on frozen section samples according to the manufacturer’s instructions. On each individual section, five target mRNAs were examined: atonal homolog1 (*Atoh1*; #574081-C2 for ggATOH1, ACD), secreted frizzled-related protein 2 (*Sfrp2*; #837131-C2 for ggSFRP2), cysteine-rich angiogenic inducer 61 (*Cyr61*; #837121-C2 for ggCYR61), ribonucleotide reductase regulatory subunit M2 (*Rrm2*; #838951-C2 for ggRRM2), and interferon-alpha inducible protein 6 (*Ifi6*; #847081-C2 for ggIFI6). Both positive and negative control probes [#453961-C2 for ggUBC (ubiquitin C) or No.320751, ACD] were also labeled with alkaline phosphatase and red substrates for control purposes. The GenBank accession numbers and probe regions for each target probe are as follows: ggATOH1 (GenBank, XM_004941130.3; probe region, 107–1545), ggSFRP2 (GenBank NM_204773.1; probe region, 64–1019), ggCYR61 (GenBank NM_001031563.1; probe region, 498–1666), ggRRM2 (GenBank XM_001231544.5; probe region, 393–1843), and ggIFI6 (GenBank NM_001001296.5; probe region, 2–476). DAPI was used to stain the cell nuclei, and the slides were imaged using a BX50 microscope (Olympus, Tokyo, Japan) to acquire 20× and 40× bright-field and fluorescence images with DAPI filter from more than three BP samples for each time point.

### Quantitative Real-Time Polymerase Chain Reaction

Total RNA for each sample was extracted pooled 10 BPs using the RNeasy Plus Micro kit (catalog # 74004, QIAGEN, Venlo, Netherlands) according to the manufacturer’s protocol. DNase I treatment was performed using spin columns. RNA was reverse-transcribed using the SuperScript III First-Strand Synthesis System (catalog #N8080234, Thermo Fisher Scientific, Waltham, MA, USA). Quantitative real-time polymerase chain reaction (qPCR) was performed using a StepOnePlus Real-Time PCR system (Thermo Fisher Scientific, Waltham, MA, USA). cDNA was amplified using the Power SYBR Green PCR Master Mix (catalog #4367659, Thermo Fisher Scientific, Waltham, MA, USA). Target gene expression was normalized to hypoxanthine phosphoribosyltransferase 1 (*Hprt1*, Hassanpour et al., 2019). cDNA from the brain tissue of post-hatched 1-day-old chickens was used to generate standard curves for each gene. Relative quantification was performed using Real-Time PCR System Software v2.0, with the 2^−ΔΔCT^ method (Thermo Fisher Scientific, Waltham, MA, USA). The following primers were used:

*Hprt1* forward, 5′-GCACTATGACTCTACCGACTATTGC-3′
*Hprt1* reverse, 5′-CAGTTCTGGGTTGATGAGGTT-3′
*Atoh1* forward, 5′-AACAACGACAAGAAGCTCTCCAA-3′
*Atoh1* reverse, 5′-GCGAGGGCGCTGATGTAG-3′
*Ifi6* forward, 5′-GCGCGATGTTTCTATGGCTT-3′
*Ifi6* reverse, 5′-TTGTCCCCAATCCAGCGTAT-3′

### RNA Sequencing

At the end of the culture period, the surrounding tissues, such as the tectorial membrane, were removed from the sensory epithelium with thermolysin treatment (500 μg/ml in medium 199, catalog #T7902, Sigma–Aldrich, St. Louis, MO, USA) at 37°C for 30 min, followed by transfer of BPs to media supplemented with 5% fetal bovine serum. Over 1.72 μg (OD260/280, 0.82–1.70 in OD230/280) of total RNA was collected from 50 cultured BPs for each experimental group.

RNA-seq analysis was performed using the Illumina NovaSeq6000, PE150 by Novogene (Tianjin, China). Approximately 6 Gb of row read sequence data were obtained for each sample, and FASTQ files were analyzed using the following procedure: After exclusion of rRNA sequences using Bowtie2 (ver. 2.1.0; Langmead and Salzberg, 2012), data were mapped to the reference genome (GRCg6a/galgal6) using the STAR program with ENCODE options (ver. 2.7.1a; Dobin et al., 2013). Gene expression values [in transcripts per million (TPM)] were then calculated using RSEM (ver. 1.3.0; Li and Dewey, 2011). Differentially expressed genes (DEGs) were determined with DEseq2 (ver. 1.8.2; Love et al., 2014) and the weighted average difference (WAD) method (Kadota et al., 2008). We employed DEseq2 for normalization for raw read counts and determination threshold for WAD analyses. Initially, we listed genes that fulfilled the following characteristics: *p* < 0.05, fold change for normalized read counts ≥1.3 or ≤1/1.3, raw read counts ≥7, and TPM ≥1 in either of the compared conditions with DEseq2. Then we set thresholds for WAD analyses to cover the listed genes (Supplementary Table 1). Obtained DEG were clustered with k-means clustering (*k* = 11), followed by translation to human homologous genes (NCBI homoloGene database) and gene ontology (GO)/pathway analysis with Metascape^2^ (date of analysis: 2020/8/27; Tripathi et al., 2015). For *k*-means clustering, we used *z*-score translated raw-read counts, and the parameter *k* was determined to reduce clusters with redundant expression profiles, starting from *k* = 12. Transcription factors (TFs) or co-factors were determined by the following GO terms: GO: 0005634 nucleus; GO: 0045892 negative regulation of transcription, DNA-templated; GO: 0045944 positive regulation of transcription from RNA polymerase II promoter’ GO: 0044212 transcription regulatory region DNA binding; and GO: 0006351 transcriptions, DNA-templated.

### Statistical Analysis

One-way analysis of variance (ANOVA) with a Tukey’s *post hoc* test or Student’s *t*-test was used to compare the counts and qPCR data between experimental groups with XLSTAT Basic (version; 2020.2.2.65342, Addinsoft Inc., New York, NY, USA). Data are expressed as mean ± standard deviation. Differences with *p* < 0.05 were considered statistically significant.

## Results

### Time Course of HC Regeneration in the BP Explants

**Figure 1:**
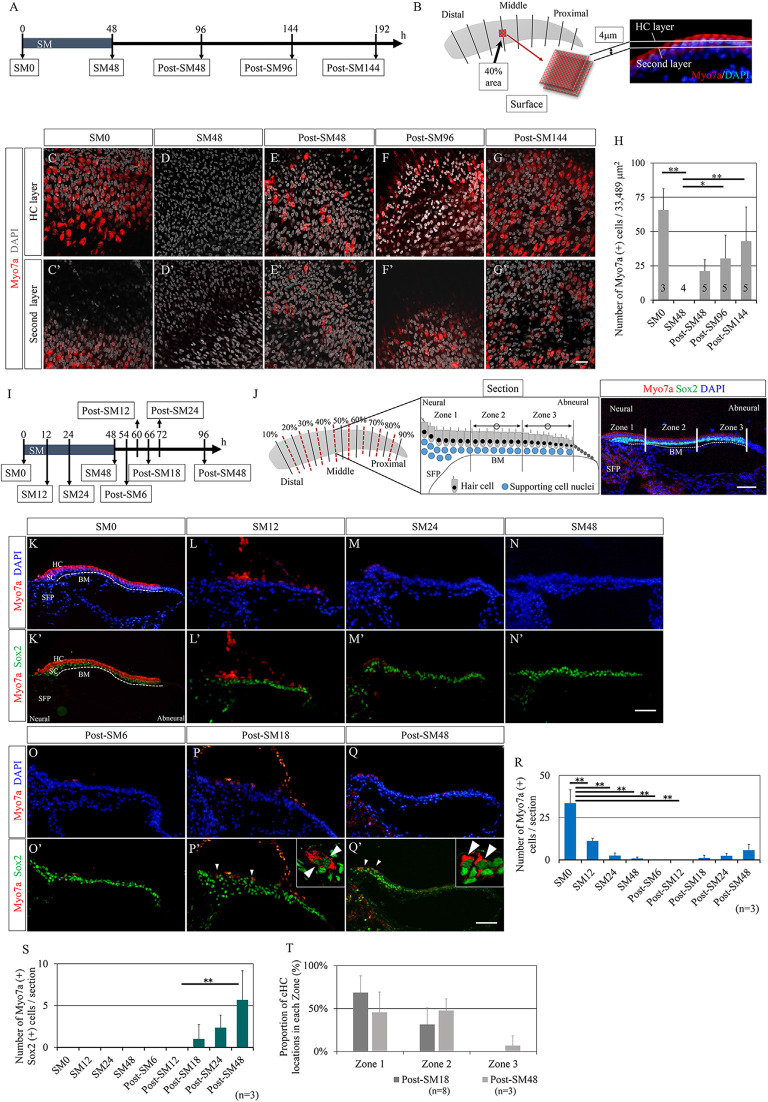
Time course of hair cell regeneration in the basilar papilla explants. **(A)** Diagram of exposure to streptomycin (SM) and sampling time points. **(B)** Schematic drawing showing the observation area and two layers for counting hair cells (HCs) that are labeled with myosin VIIa (Myo7a) and nuclear staining with 4′,6-diamidino-2-phenylindole (DAPI). **(C–G′)** Representative images of immunostaining for Myo7a (red) and nuclear staining with DAPI (gray) in two layers at each sampling time point. In SM48 samples **(D,D′)**, virtually no HCs were observed, and newly generated HCs appeared from post-SM48 **(E,E′)**. Scale bar represents 20 μm. **(H)** Myo7a-positive cells as sums of two layers in the 40% area (33,489 μm^2^). Differences in the numbers of Myo7a-positive cells between SM0 and SM48, and between SM48 and post-SM96 or post-SM144 were statistically significant [*p < 0.05, **p < 0.01, analysis of variance (ANOVA) with a Tukey’s *post hoc* test]. Bars represent standard deviations. **(I)** Diagram for sampling for the detailed analysis of HC loss and emerging new HCs. **(J)** Schematic drawing showing the preparation of serial frozen sections (red dotted lines). Three zones in a cross-section are shown in a schematic drawing as is a cross-sectional image stained for Myo7a, Sox2, and DAPI. From each sample, seven to eight sections were used for staining and quantification. The most neural zone adjacent to the superior fibrocartilaginous plate (SFP) is named Zone 1. The remaining sensory epithelia on the basilar membrane (BM) are divided equally into Zone 2 (neural side) and Zone 3 (abneural side). **(K′–Q′)** Representative images of immunostaining for Myo7a (red) and Sox2 (green) or nuclear staining with DAPI (blue). Arrowheads indicate converting HCs that are co-stained with Myo7a and Sox2. Dotted lines indicate the location of the basilar membrane (BM). The scale bar represents 50 μm. **(R)** Numbers of Myo7a-positive cells per section at each time point (**p < 0.01, ANOVA with a Tukey’s *post hoc* test). **(S)** Numbers of Myo7a and Sox2 co-stained cells per section at each time point (**p < 0.01, ANOVA with a Tukey’s *post hoc* test). **(T)** Proportions of converting hair cell locations in post-SM18 and post-SM48 samples. Bars in **(R–T)** represent standard deviations.

First, we examined the time course of HC regeneration in explant cultures of the post-hatched 1-day-old chick BP after ototoxic insult with SM. BP explants were exposed to SM for 48 h to induce HC damage, according to previous reports (Shang et al., 2010; Lewis et al., 2012, 2018). BP explants were then maintained in culture media without SM for a maximum of 144 h (Figure 1A). Samples were collected every 48 h [SM0 (before SM exposure), SM48, post-SM48, post-SM96, and post-SM144; *n* = 3–5] for histological analyses in the surface preparation (Figure 1A). HCs were identified by immunostaining for myosin VIIa and nuclear staining with DAPI. The number of HCs was manually counted in the two layers in the area 40% from the distal end of the BPs (Figures 1B–G′). Virtually no myosin VIIa-positive cells were observed in SM48 samples (Figures 1D,D′), indicating that 48 h exposure to SM caused a total loss of HCs in this region. Newly generated HCs were observed in post-SM48 samples that were incubated for an additional 48 h after SM treatment (Figures 1E,E′). Robust HCs were found in post-SM144 samples (Figures 1G,G′). Quantitative assessments revealed a significant loss of HCs in SM48 samples (*p* < 0.0001, one-way ANOVA with a Tukey’s *post hoc* test) compared with that in SM0 samples as well as a significant increase in HC numbers in post-SM96 (*p* = 0.035, one-way ANOVA with a Tukey’s *post hoc* test) and post-SM144 samples (*p* = 0.004, one-way ANOVA with a Tukey’s *post hoc* test) compared to that in SM48 samples (Figure 1H). These findings demonstrate that HC regeneration occurred in explant cultures of post-hatched 1-day old chick BPs and that newly generated HCs already appeared 48 h after SM exposure (post-SM48).

### Time Points for Total HC Loss and the Emergence of Converting HCs

Next, we aimed to clarify the exact time points at which total HC loss occurred and at which newly generated HCs appeared in chick BP explant cultures. We prepared consecutive frozen sections of BP cultures that were collected at nine time-points (*n* = 3 for each) from SM0 to post-SM48 (Figure 1I), in which newly generated HCs were identified in whole mounts (Figures 1E,E′). From each BP culture, eight sections (0–20% from the distal end, 20–30%, 30–40%, 40–50%, 50–60%, 60–70%, 70–80%, 80%-the proximal end) were generated for histological analyses (Figure 1J). In a cross-section of BPs, we divided the sensory epithelium into three regions to validate the location of regenerated HCs (Figure 1J). In the current study, we defined myosin VIIa^+^ and Sox2^+^ double-positive cells as converting HCs. Immunostaining for myosin VIIa and Sox2 demonstrated dynamic changes in cell populations of BP explants (Figures 1K–Q′). Initially, myosin VIIa^+^ cells disappeared at SM24 (Figures 1M,M′) myosin VIIa^+^ cells, co-expressing Sox2 in their nuclei, reappeared from post-SM18 (Figures 1P,P′). Cells co-expressing myosin VIIa and Sox2 had a long and narrow shape (Figures 1P′,Q′), consistent with the morphological characteristics of converting HCs (Stone and Cotanche, 2007; Daudet et al., 2009). A quantitative assessment of myosin VIIa^+^ cells demonstrated a precise time course of HC loss and regeneration (Figure 1R). A significant loss of HCs was observed at SM12 (*p* < 0.0001, one-way ANOVA with a Tukey’s *post hoc* test) compared to that at SM0. Virtually no HCs existed in BPs from SM24 to post-SM12 (Figure 1R). The number of converting HCs that expressed both myosin VIIa and Sox2 increased over time (Figure 1S).

We also examined the location of the converting HCs in BPs. Zone 1 is the most neural-sided portion of BPs, namely the neural edge, which is adjacent to the superior fibrocartilaginous plate (SFP; Figure 1J). SFP is a cartilaginous tissue that is located over the cochlear ganglion and connects with the basilar membrane. The nerve fibers between the cochlear ganglion neurons and HCs run through the SFP. The sensory epithelium on the basilar membrane was divided into two equal parts: Zone 2 (neural side) and Zone 3 (abneural side; Figure 1J). In post-SM18 samples, converting HCs were observed only in Zones 1 and 2 (Figure 1T). In post-SM48, converting HCs were also found in Zone 3, but they were predominantly located in Zone 1 or 2 (Figure 1T). Nearly total HC loss was observed at SM24, and converting HCs first appeared in the neural half of BPs in post-SM18 samples.

### Cell Division of SCs in BP Explants After HC Loss

**Figure 2:**
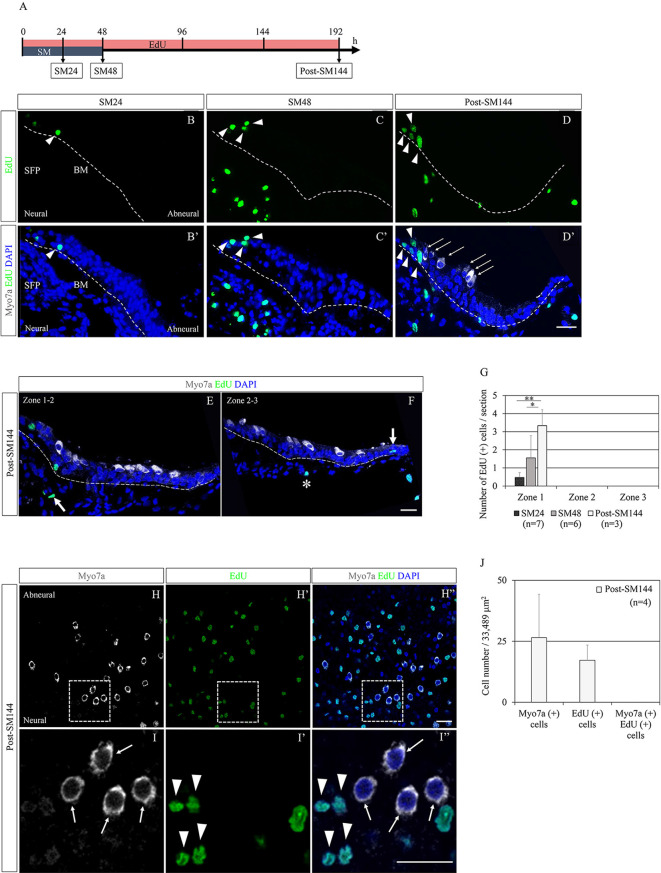
Cell division of supporting cells in basilar papilla (BP) explants after hair cell (HC) loss and its contribution to HC regeneration. **(A)** Diagram of EdU labeling assay. **(B–D′)** Representative images of EdU labeling (green; arrowheads) in cross-sections of SM24, SM48, and post-SM144 samples. Nuclear staining with 4′,6-diamidino-2-phenylindole (DAPI) is shown as blue staining, and immunostaining for myosin VIIa (Myo7a; arrows) is shown as gray. Dotted lines indicate the location of the basilar membrane. Scale bar represents 20 μm. **(E,F)** Representative images of Myo7a-positive cells in Zones 2 and 3 in cross-sections of post-SM144 samples. Nuclear staining with DAPI is shown as blue staining, and immunostaining for Myo7a is shown as gray. Dotted lines indicate the location of the basilar membrane (BM). Scale bar represents 20 μm. An arrow in **(E)** indicates EdU labeling in the superior fibrocartilaginous plate (SFP). An arrow in **(F)** indicates EdU labeling in a hyaline cell, and an asterisk indicates EdU labeling a mesenchymal cell underneath the BM. Scale bar represents 20 μm. **(G)** The number of EdU-positive cells per section in Zones 1–3 in SM24, SM48, and post-SM144 samples. All EdU-labeled cells were found in Zone 1. Differences in the number of EdU-positive cells were statistically significant between SM24 and post-SM144 (**p = 0.001 by Tukey’s *post hoc* test) and between SM48 and post-SM144 (*p = 0.028 by Tukey’s *post hoc* test). Bars represent standard deviations. **(H–I″)** Representative images of EdU labeling in post-SM144 samples in surface preparation. Upper panels **(H–H″)** are low-magnification views, and lower panels **(I–I″)** show high-magnification views of the area within the dotted squares in the upper panels. Arrowheads indicate EdU labeling, and arrows indicate Myo7a-expressing cells. Scale bars represent 20 μm. **(J)** The number of Myo7a-positive cells, EdU-positive cells, or Myo7a and EdU double-positive cells in the area 40% from the distal end of the BPs of post-SM144 samples. Bars represent standard deviations.

Mitotic division of SCs could also be involved in the restoration of BPs, although the predominant mode of HC regeneration in chick BPs is reported to be direct transdifferentiation of SCs (Stone and Cotanche, 2007; Shang et al., 2010). Scheibinger et al. (2018) proposed three different behaviors of SCs during the process of HC regeneration in chick utricles: asymmetric division, direct transdifferentiation, and symmetric division for the replenishment of SCs. To evaluate the SC division and its contribution to HC regeneration in our culture model, we performed EdU labeling. EdU was added to the culture media throughout the culture period and sample collection was performed at SM24, SM48, and post-SM144 (*n* = 3–7; Figure 2A). In SM24 samples, EdU-positive cells were found in the neural edges of BPs (Figures 2B,B′). The distribution of EdU-positive cells in SM24 samples was sporadic. In SM48 samples, EdU-positive cells were distributed in the neural edges of BPs (Figures 2C,C′). Besides, EdU-labeled cells were also found in the SFP (Figures 2C,C′). The distribution of EdU-positive cells was not sporadic at SM48. Some EdU-positive cells were adjacent to other EdU-positive cells (Figures 2C,C′). In post-SM144 samples also, EdU labeling was found in the neural edges of BPs (Figures 2D,D′). Myosin VIIa-positive cells were observed in the neural edge of BPs of post-SM144 samples, but these cells did not exhibit EdU labeling (Figures 2D,D′). In both Zones 2 and 3, several myosin VIIa-positive cells were also identified, while no EdU labeling was found in myosin VIIa-positive cells (Figures 2E,F). Quantitative assessments of EdU-positive cells in BP cross-sections were performed according to the three separate zones of BPs (Figure 1J). All EdU-positive cells were located in Zone 1, the neural edge of BPs, in SM24, SM48, and post-SM144 samples (Figure 2G). Time-dependent increases in the number of EdU-positive cells were found (*p* = 0.001 by one-way ANOVA). Differences in the number of EdU-positive cells were statistically significant between SM24 and post-SM144 (*p* = 0.001 by Tukey’s *post hoc* test) and between SM48 and post-SM144 (*p* = 0.028 by Tukey’s *post hoc* test).

To confirm the absence of myosin VIIa-positive cells labeled with EdU, we also assessed for EdU-positive cells in the surface preparation of post-SM144 samples (Figures 2H–I″). In the area, 40% from the distal end of the BPs of post-SM144 samples, 26.5 ± 17.8 myosin VIIa-expressing cells, and 17.25 ± 6.2 EdU-positive cells were observed, while EdU incorporation was not observed in any myosin VIIa-expressing cells in the surface preparation (Figure 2J). These findings strongly suggest that myosin VIIa-positive cells in post-SM144 samples were generated through direct transdifferentiation of SCs. Altogether, SC division in the neural edge of BPs was initiated immediately after total HC loss, which may contribute to the replenishment of SCs, not the increase in myosin VIIa-positive cells.

### Clustering DEGs in BPs in the Early Phase of HC Regeneration

**Figure 3:**
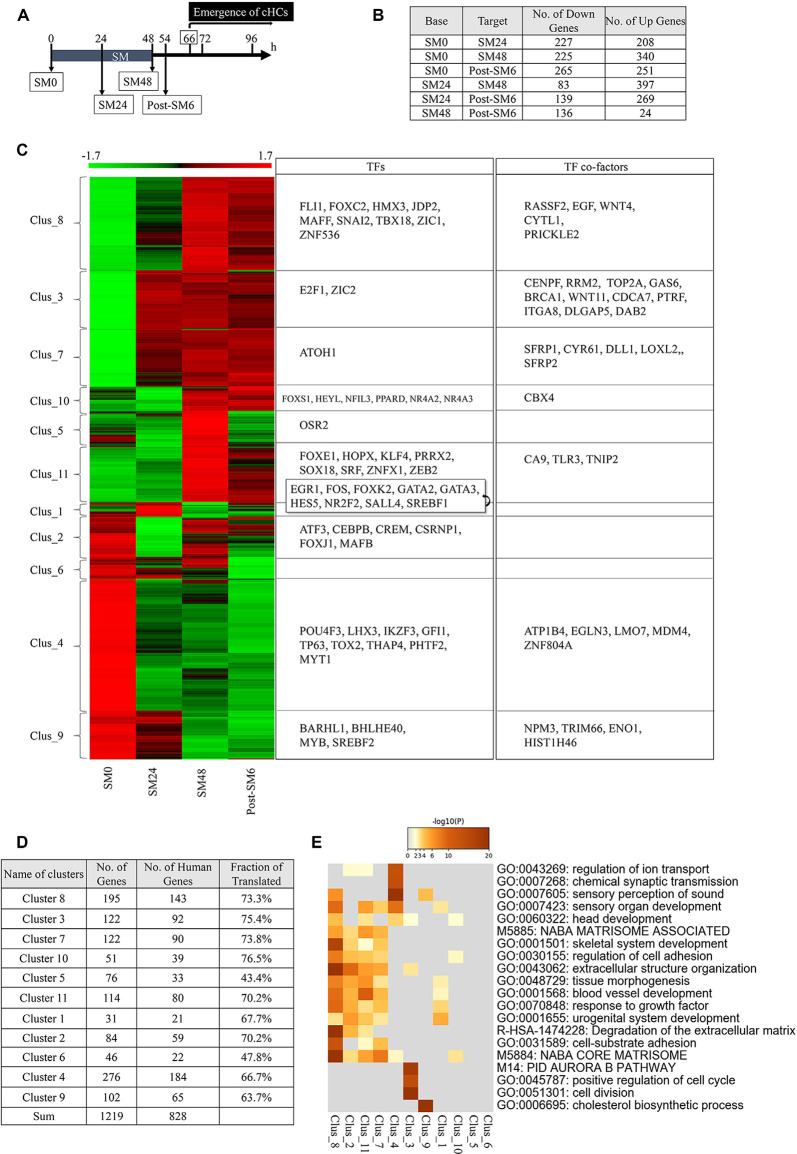
Differentially expressed genes (DEGs) in the basilar papilla (BP) explants during the initial phase of hair cell (HC) regeneration. **(A)** Diagram of sampling time points for RNA sequencing. Converting hair cells (cHCs) were observed at 66 h. **(B)** The number of differentially expressed genes (DEGs; *p* < 0.05, fold change for normalized read counts ≥1.3 or ≤1/1.3, raw read counts ≥7, and TPM ≥1 in either of the compared conditions for DEseq2 followed by the weighted average difference (WAD) method) at each time point. **(C)** Heat map showing 11 clusters determined with K-means clustering and transcription factors (TFs) or TF co-factors in each cluster. **(D)** Numbers and ratios for the human homologs of DEGs in each cluster. **(E)** Metascape GO enrichment analysis of human homologs of DEGs for each cluster.

To examine possible alterations in the expression levels of genes, we performed bulk RNA-seq of BP explants. Based on the time course of HC loss and the emergence of converting HCs in histological assessments, we set four time-points for sampling: SM0 (before SM exposure), SM24 (24-h exposure to SM, the time point for total HC loss), SM48 (48-h exposure to SM, the endpoint of SM exposure), and post-SM6 (6 h after SM treatment, 12 h before the emergence of converting HCs; *n* = 50 for each time point; Figure 3A). We found a total of 19, 238 expressed genes in these four groups.

Among the 19,238 genes expressed in BPs, 1,219 genes were identified as DEGs (*p* < 0.05, fold change ≥1.3 or ≤1/1.3, raw read counts ≥7, and TPM ≥1 in either of the compared conditions for DEseq2 followed by WAD). A remarkable number of DEGs were found between SM0 and SM24 (227 downregulated and 208 upregulated). Further changes were observed between SM24 and SM48 (83 downregulated and 397 upregulated), and between SM48 and post-SM6 (136 downregulated and 24 upregulated; Figure 3B). These findings indicate that dynamic changes occurred during and immediately after HC loss.

These 1,219 DEGs were divided into 11 clusters with K-means clustering (Figure 3C, Supplementary Table 2). Clusters 4, 6, and 9 included DEGs that were downregulated during the experimental time course (Figure 3C). Clusters 3 and 7 included upregulated DEGs from SM24, and Clusters 8, 10, and 11 included upregulated genes from SM48 (Figure 3C). Cluster 1 included DEGs showing temporal downregulation at SM48, Cluster 2 included DEGs showing temporal downregulation at SM24, and Cluster 5 included DEGs showing temporal upregulation at SM48 (Figure 3C). The human homologs of our DEGs (Figure 3D) were used to perform GO enrichment analysis using Metascape (Figure 3E). In Cluster 4, the following categories were enriched: “sensory perception of sound,” “sensory organ development,” and “chemical synaptic transmission.” Cluster 7 was characterized by “NABA_CORE_MATRISOME,” “extracellular structure organization,” “response to growth factor,” and “regulation of cell adhesion.” Clusters 2, 8, and 11 exhibited a similar trend to Cluster 7. Cluster 3 was characterized by “cell division,” “Aurora B pathway,” and “positive regulation of cell cycle.” These findings indicate that DEGs associated with the structure and function of the inner ear, especially those associated with HCs, were downregulated after SM treatment, indicating that such changes may reflect the degeneration of HCs. However, DEGs associated with tissue reorganization and cell proliferation were upregulated immediately after HC loss.

Among the 1, 219 DEGs, we identified 55 TFs and 34 TF co-factors according to GO terms (Figure 3C). TFs associated with HC differentiation was found in Cluster 9 (BARHL1), Cluster 4 (POU4F3, LHX3, GFI1), and Cluster 7 (ATOH1). Except for ATOH1, TFs associated with HC differentiation were downregulated, which may be related to the absence of HCs in SM24, SM48, and post-SM6 samples. Also, RNA-seq sampling was performed during the early phase of HC regeneration, during which converting HCs in BPs are still in the initial stage of differentiation. Therefore, HC differentiation genes may not have been upregulated during our observation period.

### Alterations in Known Signaling Pathways in the Initial Phase of HC Regeneration

**Figure 4:**
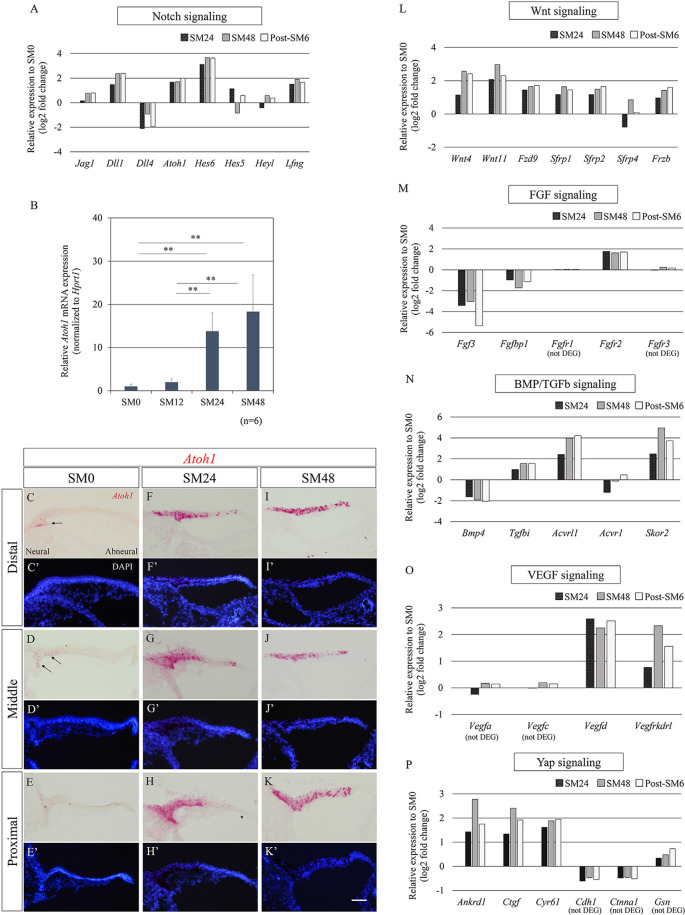
Alterations in genes associated with known signaling pathways during the initial phase of hair cell (HC) regeneration. **(A)** Relative expression levels of differentially expressed genes (DEGs; *p* < 0.05, fold change for normalized read counts ≥1.3 or ≤1/1.3, raw read counts ≥7, and TPM ≥1 in either of the compared conditions for DEseq2 followed by the WAD method) associated with Notch signaling compared to SM0 samples. **(B)** Relative mRNA expression levels of *Atoh1* by quantitative RT-PCR. *Atoh1* mRNA levels are expressed relative to *Hprt1*. **p < 0.01, ANOVA with a Tukey’s *post hoc* test. Bars represent standard deviations. **(C–K′)** *In situ* hybridization for *Atoh1* mRNA and nuclear staining with 4′,6-diamidino-2-phenylindole (DAPI) in the distal, middle, and proximal portions of the basilar papillae (BP). The intense expression of *Atoh1* was observed in SM24 and SM48 samples **(F–K′)**. Arrows indicate *Atoh1* expression in SM0 samples **(C,D)**. Scale bar represents 40 μm. **(L–P)** Relative expression levels of genes associated with Wnt **(L)**, fibroblast growth factor (FGF; **M**), bone morphogenetic protein (BMP)/Transforming growth factor-beta (TGFb; **N**), vascular endothelial growth factor (VEGF; **O**), and Yap signaling **(P)** compared to SM0 samples. Genes that did not pass our filter for differentially expressed genes are stated as (not DEG).

We attempted to illustrate trends of alterations in transcripts of known signaling pathways—Notch, FGF, Wnt, bone morphogenetic protein (BMP)/transforming growth factor-beta (TGFb), vascular endothelial growth factor (VEGF), and Yap—among the 1, 219 DEGs identified (*p* < 0.05, fold change ≥1.3 or ≤1/1.3, raw read counts ≥7, and TPM ≥1 in either of the compared conditions for DEseq2 followed by WAD). The differentially expressed levels, compared to SM0 samples, are shown in Figures 4A,L–P. Among Notch signaling-associated genes, *Jag1, Dll1, Dll4, Atoh1, Hes6, Hes5, Heyl*, and *Lfng* tended to be upregulated (Figure 4A), consistent with previous observations of HC formation in developing chick BPs (Petrovic et al., 2014) and HC regeneration in mature chick BPs (Daudet et al., 2009). Among these genes, *Atoh1* is a fundamental component for the induction of HC formation or regeneration (Lewis et al., 2012; Petrovic et al., 2014). To determine the timing and levels of *Atoh1* upregulation, we examined alterations in *Atoh1* mRNA expression by qPCR (Figure 4B). For this purpose, BP samples that were exposed to SM for 12 h were prepared, in addition to the SM0, SM24, and SM48 samples (*n* = 6 for each). In SM12 samples, no significant elevation of *Atoh1* mRNA levels was found compared to SM0 samples (*p* = 0.9999, one-way ANOVA with a Tukey’s *post hoc* test). SM24 and SM48 samples exhibited significant elevation compared to the SM0 or SM12 samples (fold change: 13.79 ± 4.24, *p* = 0.0005 for SM0 vs. SM24, fold change: 7.00 ± 2.15, *p* = 0.002 for SM12 vs. SM24, fold change: 18.30 ± 8.52, *p* < 0, 0001 for SM0 vs. SM48, fold change: 9.29 ± 4.32, *p* < 0.0001 for SM12 vs. SM48, one-way ANOVA with a Tukey’s *post hoc* test; Figure 4B). ISH for *Atoh1* demonstrated a spatial pattern of SCs showing *Atoh1* upregulation (Figures 4C–K′). In SM0 samples, *Atoh1* expression was observed in a small number of SCs on the neural edge of the distal and middle portions of the BPs (Figures 4C,D′). In SM24 and SM48 samples, intense expression of *Atoh1* mRNA was observed on both the neural and abneural sides of BPs (Figures 4F–K′), which is similar to the pattern observed in the acute phase of damaged BPs *in vivo* (Cafaro et al., 2007; Lewis et al., 2018). These findings confirmed that *Atoh1* is upregulated in SCs immediately after total HC loss.

Besides the Notch signaling pathway, we monitored alterations in genes associated with the Wnt, FGF, BMP/TGFb, VEGF, and Yap signaling pathways (Figures 4L–P). Among Wnt signaling-associated genes, expression of two Wnt ligands, *Wnt4* and *Wnt11*, one frizzled receptor, *Fzd9*, and four secreted frizzled-related Wnt inhibitors, *Sfrp1, Sfrp2, Sfrp4*, and *Frzb*, exhibited a trend of upregulation (Figure 4L). Of these seven genes, *Sfrp2*, *Frzb*, and *Fzd9* have also been found to be upregulated from the early development stage of chick BPs and contribute to the specification of BPs (Sienknecht and Fekete, 2008). Among FGF signaling-related genes, *Fgfr2* was upregulated, while *Fgf3* and *Fgfbp1* were downregulated (Figure 4M). The expression of *Fgfr1* and *Fgfr3*, which are known to be expressed in the chick BP (Bermingham-McDonogh et al., 2001; Jacques et al., 2012a; Honda et al., 2018), exhibited no alteration (Figure 4M). Among BMP/TGFb signaling-associated genes, downregulation of *Bmp4* was observed (Figure 4N), which may be a consequence of HC loss as described previously (Lewis et al., 2018). Upregulation of *Tgfbi*, TGFb signaling downstream, and *Acvrl1* and *Acvr1*, type 1 receptors of TGFb family ligands were observed, while *Skor2*, a TGFb antagonist was also upregulated (Figure 4N). In VEGF signaling, *Vegfd* and *Vegfrkdrl*, a VEGF signaling ligand and its receptor, were upregulated (Figure 4O), suggesting the activation of VEGF signaling in BPs. Three major YAP signaling downstream genes, *Ankrd1*, *Ctgf*, and *Cyr61*, were upregulated, while genes encoding inhibitors of Yap translocation to the nucleus, *Cdh1*, *Ctnna1*, and *Gsn*, were detectable, but not passed our filter for DEGs (Figure 4P), indicating the involvement of Yap signaling in the initial phase of HC regeneration in chick BPs.

Taken together, *Atoh1* upregulation immediately after total HC loss was observed in our explant culture model, similar to previous observations in chick BPs (Cafaro et al., 2007; Lewis et al., 2018). A trend showing upregulation of *Dll1* and *Hes6*, which are thought to be downstream of Atoh1 (Mulvaney and Dabdoub, 2012), suggested that the differentiation process to HCs had already been initiated in *Atoh1*-expressing SCs at SM24. Alterations in major signaling pathways associated with HC formation or regeneration suggested involvements of these signaling pathways in the early phase of HC regeneration in our culture model consistent with previous observations.

### Expression Patterns of Upregulated Genes in the Early Phase of HC Regeneration

To explore the molecular events associated with the responses in SCs during initiation of HC regeneration, we focused on Clusters 3 and 7 that included DEGs upregulated from SM24. We chose two TF co-factors, *Sfrp2* (secreted frizzled-related protein 2) and *Cyr6*1 (cysteine-rich angiogenic inducer 61) from Cluster 7 and one TF co-factor, *Rrm2* (ribonucleotide reductase regulatory subunit M2) from Cluster 3 as the DEGs of interest (Figure 3C). SFRP2 is a soluble Wnt inhibitor that is expressed in developing BPs (Sienknecht and Fekete, 2008). CYR61 or cellular communication network factor (CCN) 1 is a member of the CCN family of matricellular proteins, and one of the Yap targets (Gnedeva et al., 2017). RRM2 is expressed during the late G1/early S phase when DNA replication occurs (Chabes et al., 2003, 2004).

**Figure 5:**
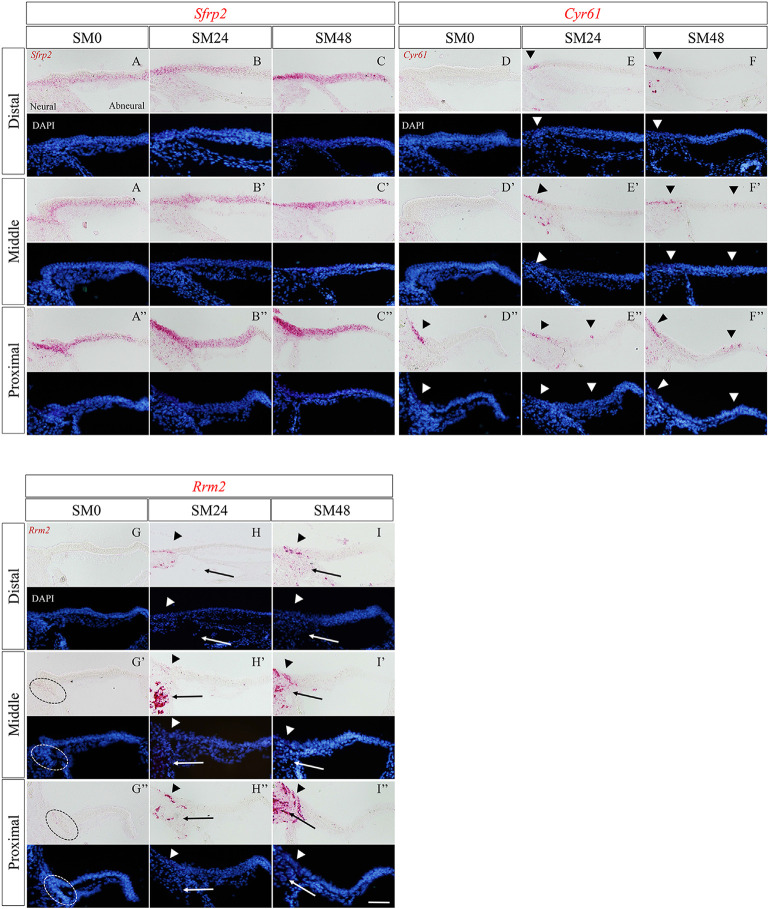
The expression patterns of the newly identified genes in the basilar papilla (BP) in the early phase of hair cell (HC) regeneration. **(A–I″)** Representative images of *in situ* hybridization for *Sfrp2* **(A–C″)**, *Cyr61* **(D–F″)**, and *Rrm2* **(G–I″)** in BPs of SM0, SM24, and SM48 samples. Cells were counterstained with 4′,6-diamidino-2-phenylindole (DAPI). Arrowheads indicate the location of expressed cells in the BP (red). Dotted lines in **(G′,G″)** indicate *Rrm2* expression in SCs in the neural edge of SM0 samples. Arrows in **(H–I″)** indicate *Rrm2* expression in cells in the superior fibrocartilaginous plate (SFP). Scale bars represent 40 μm.

Expression of *Sfrp2* in SM0 samples was observed in SCs, with broad distribution (Figures 5A–A″). The expression pattern of *Sfrp2* in SM0 samples was identical to that in the late embryonic stage of chick BPs (Sienknecht and Fekete, 2008). In SM24 and SM48 samples, *Sfrp2* expression was intense in SCs, especially in the neural half of BPs (Figures 5B–C″). No alteration in the distribution of *Sfrp2* was observed.

In SM0 samples, virtually no expression of *Cyr61* was observed in SCs in the distal or middle portion of BPs (Figures 5D,D′). *CYR 61* expression was found around the neural edge of BPs in the proximal portion (Figure 5D″). In SM24 and SM48 samples, intense expression was observed in and/or outside of the neural edges of BPs (Figures 5E–F″). Some SCs in the abneural portion of BPs showed *Cyr61* expression (Figures 5E″,F′,F″).

In SM0 samples, weak expression of *Rrm2* was observed in the neural edge of the middle and proximal portions (Figures 5G′,G″). In SM24 and SM48 samples, strong expression of *Rrm2* was found in and/or outside of the neural edge of BPs (Figures 5H–I″). Also, *Rrm2* expression was observed in the cells that were present in SFP (Figures 5H,I″). The distribution of *Rrm2* expression in SM24 and SM48 samples was identical to that of EdU-positive cells in SM48 samples (Figures 2C,C′).

### Alterations in Type I Interferon Signaling-Associated Genes

To explore candidate signals for initiating activation of SCs after HC loss, we sought DEGs that were upregulated at SM24 compared to SM0 and downregulated at SM48 or post-SM6 compared to SM24. Consequently, only one DEG, IFI6, was identified in Cluster 3. *IFI6* is a type I interferon (IFN)-stimulated gene that is regulated by the JAK/ STAT signaling pathway (Friedman et al., 1984; Porter et al., 1988). The JAK/STAT signaling pathway is a key regulator of HC regeneration in zebrafish lateral lines (Liang et al., 2012). Bulk RNA-seq analysis of HC regeneration in chick BPs *in vivo* also demonstrated the involvement of JAK/STAT signaling (Jiang et al., 2018). Hence, we focused on type I IFN/JAK/STAT signaling.

**Figure 6:**
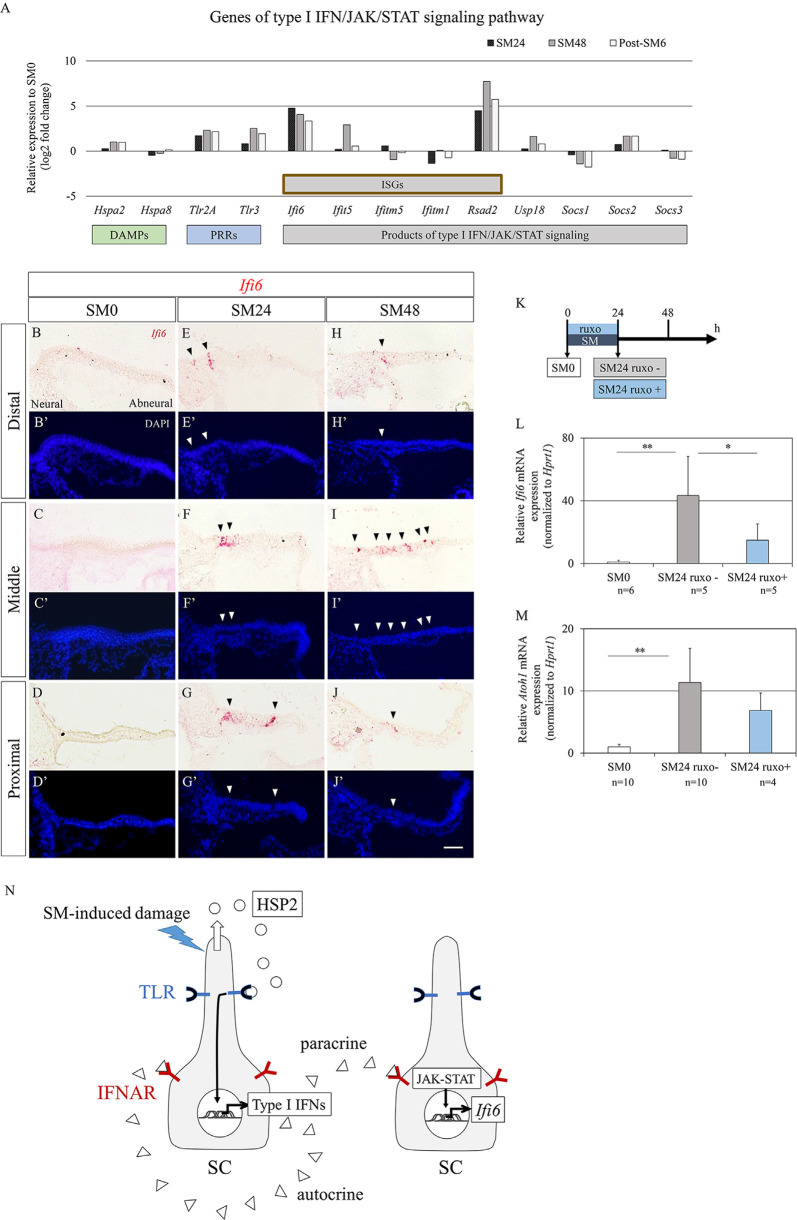
Alterations in genes associated with type I interferon (IFN) signaling in basilar papilla (BP) explants. **(A)** mRNA expression levels (log_2_ fold changes) of differentially expressed genes (*p* < 0.05, fold change for normalized read counts ≥1.3 or ≤1/1.3, raw read counts ≥7, and TPM ≥1 in either of the compared conditions for DEseq2 followed by the WAD method) associated with type I IFN/JAK/signal transducer and activator of transduction (STAT) signaling pathway relative to their expression levels at SM0. **(B–J′)** Representative images of *in situ* hybridization for *Ifi6* mRNA expression and nuclear staining with 4′,6-diamidino-2-phenylindole (DAPI) in the BP. Arrowheads indicate *Ifi6* mRNA expression. Scale bar represents 40 μm. **(K)** Diagram of exposure to streptomycin (SM) and ruxolitinib phosphate (ruxo) and sampling time points. **(L,M)** Relative mRNA expression levels of *Ifi6* **(L)** and *Atoh1* **(M)** by quantitative RT-PCR. *Ifi6* mRNA levels are expressed relative to *Hprt1* (**p < 0.01, *p < 0.05 by Student’s *t*-test). Bars represent standard deviations. **(N)** Hypothetical model of type I IFN/JAK/STAT signaling regulation in chick BPs. Streptomycin (SM)-induced damages induce the secretion of heat shock protein 2 (HSP2) family from supporting cells (SCs), which activates Toll-like receptors (TLR) on SCs resulting in the production of type I IFNs. Type I IFNs activate interferon alpha and beta receptor subunits (IFNAR) on SCs in a paracrine or autocrine fashion leading to upregulation of IFN-stimulated genes including *Ifi6* through JAK/STAT signaling.

Type I IFN/JAK/STAT signaling-associated genes are divided into three groups: type I IFN production, type I IFN receptors, and JAK/STAT signaling mediators of type I IFN/JAK/STAT signaling. Type I IFNs are secreted after recognition of damage-associated molecular patterns (DAMPs) and pathogen-associated molecular patterns by pattern recognition receptors (PRRs). We found 13 DEGs encoded molecules associated with the type I IFN/JAK/STAT signaling pathway (Figure 6A). As candidates of DAMPs, heat shock protein A family genes, *Hspa2* and *Hspa8* were identified. As *Hspa8* showed a trend of downregulation, *Hspa2* can be a candidate of DAMPs in our culture model. Among genes encoded PRRs, a trend for upregulation was observed in *Tlr2a* and *Tlr3* (Figure 6A). In downstream of type I IFN/JAK/STAT signaling, six IFN-stimulated genes (ISGs), and *Usp18*, which are included products of non-canonical JAK/STAT pathway, and three genes that are products of canonical JAK/STAT pathway were found in DEGs (Figure 6A). Two canonical JAK/STAT pathway-associated genes, *Socs1* and *Socs3* showed a trend of downregulation. As for type I IFNs and their receptors, *Ifnal2*, *Ifnal6, Ifnar1, and Ifnar2* were detectable in each time point, but did not pass our filter for DEGs. To confirm the alteration in *Ifi6* expression in BPs, we performed ISH (Figures 6B–J′). Virtually no expression was found in SM0 samples (Figures 6B–D′), while both the SM24 and SM48 samples exhibited intense expression in some SCs (Figures 6E–J′). SCs expressing *Ifi6* exhibited a scattered distribution in BPs and were frequently found in the neural half of BPs (Figures 6E–J′). These findings indicate the activation of non-canonical type I IFN/JAK/STAT signaling in SCs of damaged BPs.

To examine an effect of JAK/STAT signaling on *Atoh1* activation in SCs, we performed an experiment using pharmacological inhibition of JAK/STAT signaling (Figure 6K). Chick BP explants were cultured with SM and a JAK inhibitor, ruxolitinib phosphate for 24 h. Assessments by qPCR demonstrated a significant increase of *Ifi6* expression in SM24 samples that were incubated with SM alone compared with SM0 samples (*p* = 0.002 by Student’s *t*-test; Figure 6L). Supplementation of ruxolitinib phosphate significantly attenuated *Ifi6* upregulation at 24 h after SM exposure (*p* = 0.0459 by Student’s *t*-test; Figure 6L), indicating that a JAK inhibitor, ruxolitinib phosphate efficiently blocked JAK-STAT signaling in our explant cultures. The expression level of *Atoh1* was also increased in SM24 samples in comparison with SM0 samples (*p* < 0.0001 by Student’s *t*-test; Figure 6M). Ruxolitinib phosphate treatment showed a trend to decrease *Atoh1* expression levels in comparison with SM24 samples, although the difference was not statistically significant (*p* = 0.150 by Student’s *t*-test; Figure 6M). These findings suggested that IFN/JAK/STAT signaling could be associated with *Atoh1* activation in SCs of chick BPs in response to SM-induced damage.

## Discussion

In the current study, we established a new chick BP model for HC regeneration using explant cultures. In our model, the generation of new HCs mainly occurred through direct transdifferentiation of SCs. Known molecular events in the initial phase of HC regeneration were also observed in our model. In particular, alterations in *Atoh1* expression observed in the present study are consistent with other similar observations in the early phase of HC regeneration in chick BPs (Cafaro et al., 2007; Lewis et al., 2012). Bulk RNA-seq during the initial phase of HC regeneration indicated several unique transcriptional responses associated with SC activation. Taken together, our BP explant culture model can be utilized to explore the mechanisms of SC activation in the initial phase of HC regeneration.

### Two Different Responses of SCs During HC Loss

In addition to direct transdifferentiation of SCs, the mitotic division of SCs, another response of SCs after induction of HC loss, was also observed during the initial phase of HC regeneration in our model. However, myosin VIIa-positive cells did not show EdU incorporation during the culture period. These results strongly suggest that the mitotic division of SCs contributes to the replenishment of SCs in our model. However, it is also possible that SCs after mitotic division disappeared due to cell death during differentiation into HCs, or that several SC divisions resulted in EdU dilution to undetectable levels in newly generated HCs. Shang et al. (2010) demonstrated that a high concentration of bromodeoxyuridine, a traditional thymidine analog, affected HC differentiation of SCs incorporated bromodeoxyuridine, although it increased the labeling ratio of proliferating SCs, suggesting that EdU incorporation could prevent HC differentiation of SCs. Hence, EdU labeling used in the present study can affect SC differentiation into HCs. In present results, EdU labeling was limitedly observed in the neural edge of BPs, even in post-SM144 samples (Figures 2D–G), suggesting that SC division occurred only in the neural edge of BPs in our explant culture model. We assessed the expression of *Rrm2*, which is one of the DEGs identified by RNA-seq in the present study and is a marker for the late G1/early S phase of the cell cycle (Chabes et al., 2003, 2004). The results showed a similar distribution of *Rrm2* expression and EdU labeling (Figures 5G–I″), which supports our hypothesis that SC division occurred only in the neural edge of BPs. On the other hand, expression of *Atoh1* after HC loss was mainly observed in the neural half of BPs (Figures 4C–K′), indicating that SC activation in response to HC loss occurred in broader areas than did SC division. Also, converting HCs initially appeared in the neural half of BPs (Figures 1P′,Q′,T). These results indicate that two different responses of SCs, direct transdifferentiation to HCs and mitotic division, were induced simultaneously in the initial phase of HC regeneration. In the neural edge of BPs, two different types of SCs may be present based on their proliferative ability and cell fate.

### Alterations in Known Signaling Pathways in the Early Phase of HC Regeneration

In the current study, we examined changes in the expression patterns of genes associated with the Notch, Wnt, FGF, BMP/TGFb, VEGF, and Yap signaling pathways in the initial phase of HC regeneration in our explant culture model. Previous studies have revealed the critical roles of these signaling pathways in the development and regeneration of auditory sensory epithelia (Schimmang, 2007; Atkinson et al., 2015; Ebeid and Huh, 2017; Gnedeva et al., 2017, 2020; Denans et al., 2019; Samarajeewa et al., 2019; Rudolf et al., 2020; Wan et al., 2020). Also, cross-talk between these signaling pathways has been reported (Millimaki et al., 2007; Sweet et al., 2011; Jacques et al., 2012b; Munnamalai and Fekete, 2016; Lewis et al., 2018).

Previous studies demonstrated that *Atoh1* was upregulated in SCs in two different phases of HC regeneration in chick BPs (Cafaro et al., 2007; Lewis et al., 2012). In the acute phase of damage, activated SC progenitors expressed *Atoh1*, which showed a broad distribution in BPs, in contrast with the restricted *Atoh1* expression in the later phase of SC differentiation into HCs (Cafaro et al., 2007). The upregulation of *Atoh1* observed in the present study is likely associated with the activation of SCs in response to HC loss, not with lateral inhibition associated with HC differentiation, because of other HC differentiation genes (*Pou4f3, Lhx3, Gfi1*, and *Barhl1*) were downregulated. On the other hand, a trend for the upregulation of *Dll1, Hes6*, and *Lfng* was observed in the present study, which is also consistent with previous findings in the acute phase of HC regeneration in chick BPs (Daudet et al., 2009). These results indicate that the process of SC differentiation into HCs has already been initiated. Taken together, the fate of some activated SCs was already determined as direct transdifferentiation to HCs, but the process of differentiation into HCs was still in the early phase during our observation period. A recent study demonstrates that transient activation of Notch signaling induces reprogramming of adult cochlear SCs in mice, leading to the enhancement of SC potential for transdifferentiation into HCs (Shu et al., 2019). As shown in the present and previous studies (Daudet et al., 2009), Notch signaling is upregulated in chick BP SCs before direct conversion to HCs. This indicates that manipulation of the pathways identified in the present study could affect the potential of mature mammalian SCs for transdifferentiation into HCs.

During the development of the inner ear in chicks, Wnt ligands mostly originate from non-sensory tissue domains, whereas the sensory primordia preferentially express frizzled receptors and/or secreted frizzled-related Wnt inhibitors (Sienknecht and Fekete, 2008). In the present study, a trend for the upregulation of secreted frizzled-related Wnt inhibitors, *Sfrp2* and *Frzb*, and a Frizzled receptor, *Fzd9*, was observed. These three genes are associated with the specification of BP during the early phase of development (Sienknecht and Fekete, 2008). In the late stage of development, *Sfrp2* is expressed in SCs in BPs (Sienknecht and Fekete, 2008). In the present study, ISH demonstrated that *Sfrp2* is expressed in SCs of BPs before SM exposure, consistent with a previous observation (Sienknecht and Fekete, 2008). After induction of total HC loss, *Sfrp2* expression increased, while no change in its distribution was observed. These results suggest the involvement of Wnt signaling in SC activation in the initial process of HC regeneration, similar to the development of chick BP HCs. However, further studies are required to reveal the distinct roles of these Wnt-associated genes in HC regeneration in BPs.

Among genes associated with FGF signaling, *Fgf3* downregulation was observed in the present study, which is reported in the process of HC regeneration in the lateral line neuromast in the zebrafish (Lush et al., 2019). In developing chick BP explants, pharmacological inhibition of FGF signaling results in the induction of direct SC transdifferentiation into HCs (Jacques et al., 2012a). Therefore, the *Fgf3* downregulation observed in the present study may explain the promotion of direct transdifferentiation of SCs. On the other hand, in the zebrafish lateral line, *Fgfr2* is also downregulated after HC loss (Lush et al., 2019), while in the present study, it was upregulated after HC loss. There are several differences in the roles of FGF signaling in HC formation and regeneration between species (Atkinson et al., 2015). The distinct roles of FGF signaling in the initiation of HC regeneration in chick BPs remain unclear, and further investigations are required.

A recent publication using a similar BP explant culture model to the present study demonstrated the involvement of VEGF signaling in the induction of SC proliferation in chick BPs (Wan et al., 2020). In the present study, the upregulation of VEGF signaling ligand and receptor was also found, suggesting that VEGF signaling is also related to SC proliferation observed in the present study. As upstream of VEGF signaling, a previous study (Hori et al., 2010) indicated possible roles of prostaglandin E signaling, which demonstrated that prostaglandin E induced VEGF secretion from mouse cochlear explants. Some genes encoded prostaglandin E synthase was detectable, but not passed our filter for DEGs, in the present data set of RNA-seq (Supplementary Table 2).

Recently, a couple of publications showed significant roles of Yap signaling in HC regeneration in the mouse utricle (Gnedeva et al., 2017; Rudolf et al., 2020) and the cochlea (Gnedeva et al., 2020). We identified the upregulation of Yap targets in our data set. Also, no upregulation in genes encoded inhibitors for Yap translocation into the nucleus was observed in the present study, suggesting that Yap signaling may also work in HC regeneration in chick BPs.

### Newly Identified Genes Involved in the Early Phase of HC Regeneration

In the current study, we attempted to explore novel molecules involved in the initial process of HC regeneration using our explant culture model. Bulk RNA-seq of BP explant cultures during the initial phase of HC regeneration indicated the involvement of *Cyr61* and *Rrm2*, in the initiation of SC activation. Expressions of both *Cyr61* and *Rrm2* were upregulated in BPs after total HC loss in a specific region, the neural edge of BPs (Figures 5D–I″), where EdU labeling was specifically observed (Figures 2C–D″). CYR 61 is a CCN matricellular protein that regulates diverse cellular functions, including cell adhesion, migration, proliferation, differentiation, and survival in a cell-type- and context-dependent manner (Chen and Lau, 2009). Therefore, CYR61 may affect the induction of cell proliferation, particularly on SCs in the neural edge of BPs. Also, *Cyr61* expression around the neural edge of BPs suggests a possible involvement of Yap signaling in cell proliferation in this region. RRM2 is also expressed in retinal progenitor (Trimarchi et al., 2008) and neural progenitor cells (Habib et al., 2016). CCN proteins are reported to shape the microenvironment associated with fate determination of stem or progenitor cells (Chen and Lau, 2009; Zuo et al., 2010; Lukjanenko et al., 2019). Therefore, *Cyr61* and *Rrm2* expression in the neural edge of BPs in SM0 samples (Figures 5D″,G′,G″) may indicate the presence of progenitor-like cells in this specific location. Taken together, SCs in the neural edge of BPs may have different cell characteristics from SCs in other locations.

Samarajeewa et al. (2018) demonstrated that SC proliferation by Wnt activation in the early neonatal mouse cochlea is correlated with unique transcriptional responses that diminish with age. The upregulation of *Cyr61* and *Rrm2* occurs with such transcriptional changes, suggesting the involvement of these genes in the induction of SC proliferation in the neonatal mouse cochlea (Samarajeewa et al., 2018). Therefore, SC proliferation in the neonatal mouse cochlea and chick BP after damage may share the same signaling pathways. On the other hand, *Sfrp2* was downregulated in neonatal mouse cochleae after Wnt activation (Samarajeewa et al., 2018), in contrast to the present findings. Based on the spatial and temporal expression of *Sfrp2* in damaged chick BPs, *Sfrp2* may be associated with the differentiation of activated SCs rather than induction of SC division.

### Possible Role of Type I IFN/JAK/STAT Signaling in the Initial Phase of HC Regeneration

In the current study, we focused on *IFI6*, one of the Cluster 3 genes, because it was the only gene upregulated at SM24 compared to SM0 and downregulated at post-SM6 compared to SM24. IFI*6* is a type I IFN-stimulated gene that is regulated by the JAK/STAT signaling pathway (Friedman et al., 1984; Porter et al., 1988). Hence, we examined alterations in genes associated with the type I IFN/JAK/STAT signaling pathway and identified the upregulation of several genes associated with this signaling pathway. In present results, several targets of the non-canonical type I IFN/JAK/STAT signaling pathway showed a trend for upregulation, while those of the canonical signaling pathways exhibited a trend of downregulation. ISH demonstrated the expression of *Ifi6* in SCs in damaged BPs. These findings strongly suggested the involvement of non-canonical type I IFN/JAK/STAT signaling in the initial step for HC regeneration. Pharmacological inhibition of JAK-STAT signaling exhibited significant attenuation of *Ifi6* expression and a trend for attenuation of *Atoh1* activation. Altogether, non-canonical type I IFN/JAK/STAT signaling could be associated with the activation of some populations of SCs immediately after HC loss. In neural stem cells, IFN signaling induces dormant neural stem cell subpopulations to enter the primed state in response to ischemic injury (Llorens-Bobadilla et al., 2015). Therefore, the non-canonical type I IFN/JAK/STAT signaling could initiate alterations of SC states from dormant or quiescent to primed or activated.

The primary step of type I IFN/JAK/STAT signaling is the recognition of DAMPs by PRRs. There are two candidates for DAMPs in the initiation of this signaling in chick BP explant cultures, HC debris and heat shock protein A family proteins. SCs in vestibular epithelia have phagocytic activity in response to HC death (Bird et al., 2010; Monzack et al., 2015; Hirose et al., 2017). However, published studies suggest that nearly all HC debris is extruded from the luminal surface of BPs (Hirose et al., 2004; Warchol et al., 2012) and that little or no HC debris is detected in SCs in BPs (Warchol et al., 2012), indicating that phagocytosis of SCs may not be a mechanism for the initiation of type I IFN/JAK/STAT signaling in chick BPs. Rather than phagocytosis, the recognition of heat shock protein A family proteins as DAMPs through toll-like receptors (TLR) can be a mechanism for IFN production in chick BPs (Figure 6N). Upregulation of genes encoded heat shock protein A family proteins and TLR including *Tlr2a* was found in our data set. Also, the upregulation of HSP70 was found in mouse cochlear SCs after noise exposure (Gratton et al., 2011), and expression of adaptor proteins of type I IFN/JAK/STAT signaling in the mouse cochlea has been reported (Hayashi et al., 2013). HSP70 secreted from SCs contributes to the protection of HCs against aminoglycoside toxicity in mouse utricles (May et al., 2013).

Resident macrophages in the inner ear could be associated with type I IFN/JAK/STAT signaling in chick BPs. Migration of macrophages was observed in chick BP explants after HC injury (Warchol et al., 2012), suggesting a certain role of macrophages in processing dying HCs and/or in consecutive repair processes. However, migrated macrophages are located underneath the basilar membrane of chick BPs, and elimination of macrophages does not affect the capacity for HC regeneration in chick BP explants (Warchol et al., 2012). We, therefore, presume that SCs may play a central role in IFN production in response to SM injury in chick BPs. It may be worthy to investigate the roles of TLR in responses to SM-induced damage in chick BPs.

### Comparisons of Changes in Gene Expressions Between Chick BP and Utricle Explant Cultures

To illustrate the differences and similarities in the early phase of HC regeneration between the chick BP and utricle, we referred to the data set GSE57134_CU_REGEN.txt.gz (Ku et al., 2014), in which bulk RNA-seq was performed to investigate HC regeneration processes in chick utricle explant cultures. For comparison between BP and utricle explants, we extracted the data of the early time-points from GSE57134_CU_REGEN.txt.gz, and compared changes of gene expression patterns between two time-points corresponding to SM24 and SM48 in the present study. Gene expression patterns in Notch and FGF signaling in the utricle showed similar trends to those of BPs observed in the present study. In other signaling pathways, we found two differences between BPs and utricles. One is changes in the expression levels of cell-cycle associated genes (*Cyr61* and *Rrm2*). They were saturated at SM24 in BPs in present results, while in the utricle, further remarkable increases were observed at SM48. This may be reflected the difference in the capacity for cell proliferation between two organs. Another is downstream of IFN/JAK/STAT signaling. In the BP, target genes of the non-canonical pathway were upregulated, while canonical pathway-downstream genes, *Socs1* and *Socs3*, showed a trend to increase in the utricle. Further studies are required to conclude this issue.

## Conclusions

We established a chick BP explant culture model for HC regeneration. In our model, SC behaviors were divided into direct transdifferentiation to HCs and mitosis, indicating the heterogeneity of SCs in chick BPs. Transcriptomics at the single-cell level and/or Spatio-temporal transcriptomic analysis is required to clarify how SCs are activated after HC loss. Further, the present results indicate the involvement of type I IFN/JAK/STAT signaling in the initial step of HC regeneration in chick BPs. We believe that our explant culture model will contribute to exploring the molecular mechanisms of SC activation towards HC regeneration in chick BPs and the development of therapeutics for HC regeneration in the mammalian cochlea.

## Data Availability Statement

The datasets presented in this study can be found in online repositories. The names of the repository/repositories and accession number(s) can be found below: https://www.ncbi.nlm.nih.gov/, GSE154375.

## Ethics Statement

The animal study was reviewed and approved by Animal Research Committee of Kyoto University Graduate School of Medicine.

## Author Contributions

TN, TK, MM, and NY designed the study. MM and TK performed the laboratory experiments. RY, TO, KO, SS, and NY contributed to data analysis and critical discussion. MM, TK, and TN wrote the manuscript. All authors contributed to the article and approved the submitted version.

## Conflict of Interest

The authors declare that the research was conducted in the absence of any commercial or financial relationships that could be construed as a potential conflict of interest.

## Footnotes

^1^https://imagej.nih.gov/ij/

^2^https://metascape.org/

## Supplementary Material

SUPPLEMENTARY TABLE 1 Thresholds for the weighted average difference (WAD) method.

SUPPLEMENTARY TABLE 2 Transcripts per million (TPM) and row counts of expressed genes in chick basilar papilla explants at each time point.

## Abbreviations

ANOVA: analysis of variance
BMP: bone morphogenetic protein
BP: basilar papilla
CCN: cellular communication network factor
DAMP: damage-associated molecular pattern
DAPI: 4^′^,6-diamidino-2-phenylindole
DEG: differentially expressed gene
EdU: 5-ethynyl-2′-deoxyuridine
FGF: fibroblast growth factor
GO: gene ontology
HC: hair cell
IFN: interferon
ISH: *in situ* hybridization
JAK: Janus kinase
PBS: phosphate-buffered saline
PRR: pattern recognition receptor
qPCR: quantitative real-time polymerase chain reaction
RNA-seq: RNA sequencing
SC: supporting cell
SFP: superior fibrocartilaginous plate
SM: streptomycin
STAT: signal transducer and activator of transduction
TF: transcriptional factor.
